# Hemodynamic depression after carotid surgery: Incidence, risk factors and outcomes

**DOI:** 10.1016/j.clinsp.2022.100090

**Published:** 2022-09-08

**Authors:** Lauro A.C. Bogniotti, Marcelo P. Teivelis, Francisco A.M. Cardozo, Bruno Caramelli, Nelson Wolosker, Pedro Puech-Leão, Nelson De Luccia, Daniela Calderaro

**Affiliations:** a Anesthesiology Division, Hospital das Clínicas, Faculdade de Medicina, Universidade de São Paulo (HCFMUSP), São Paulo, SP, Brazil; b Vascular and Endovascular Surgery, Hospital Israelita Albert Einstein, São Paulo, SP, Brazil; c Unidade de Medicina Interdisciplinar em Cardiologia, Instituto do Coração, Hospital das Clínicas, Faculdade de Medicina, Universidade de São Paulo (HCFMUSP), São Paulo, SP, Brazil; d Vascular Surgery Division, Hospital das Clínicas, Faculdade de Medicina, Universidade de São Paulo (HCFMUSP), São Paulo, SP, Brazil

**Keywords:** Carotid endarterectomy, Carotid stenosis, Hypotension, Carotid artery stenting

## Abstract

**Introduction:**

Hemodynamic Depression (HD) characterized by hypotension and bradycardia is a complication of carotid surgery due to direct autonomic stimulation in the carotid sinus. The authors believe the incidence of HD is high and possibly related to major cardiac complications.

**Methods:**

Analysis of patient records during admissions for carotid surgery between January 2014 and December 2018 in two hospitals. HD was defined as bradycardia or hypotension in the first 24 postoperative hours. Bradycardia was defined as heart rate < 50bpm; hypotension as systolic blood pressure < 90 mmHg, continuous use of vasopressors, or a drop in SBP > 20% compared to preoperative values. Myocardial infarction, stroke, and cardiovascular death were defined as adverse events.

**Results:**

Overall, 237 carotid surgeries (178 endarterectomies, 59 angioplasties) were studied, and the global incidence of HD was 54.4% (hypotension in 50.2%, bradycardia in 11.0%, and hypotension and bradycardia in 6.8%). The independent predictors of HD were asymptomatic carotid stenosis (OR = 1.824; 95% CI 1.014–3.280; p = 0.045), endovascular surgery (OR = 3.319; 95% CI 1.675–6.576; p = 0.001) and intraoperative hypotension or bradycardia (OR = 2.144; 95% CI 1.222–3.762; p = 0.008). Hypotension requiring continuous vasopressor infusion was the only factor independently associated with adverse cardiovascular events (OR = 5.504; 95% CI 1.729–17.529; p = 0.004).

**Discussion/conclusion:**

Incidence of Hemodynamic Depression after carotid surgery is high and independently associated with surgical technique, symptomatic repercussion of the carotid stenosis, and intraoperative hypotension or bradycardia. Hypotension requiring the continuous infusion of vasopressors was independently associated with the occurrence of MACE.

## Highlights

• Incidence of Hemodynamic Depression was 54.4% in 237 carotid surgeries.
• Asymptomatic carotid stenosis and endovascular surgery were predictors of HD.
• Hypotension requiring continuous vasopressor infusion was associated with MACE.

## Introduction

Atherosclerotic extracranial cerebrovascular disease is a major cause of stroke, and a leading cause of death,^1^ accounting for 6.3 million or 11.8% of all lost lives worldwide in 2015.^2^ In the effort to prevent stroke, surgical therapies such as Carotid Endarterectomy (CEA) and Carotid Artery Angioplasty and Stenting (CAS) were developed to complement clinical treatment in patients at risk.^3^, ^4^, ^5^

Among arterial surgeries, carotid revascularization has a lower risk of cardiovascular events than aortoiliac procedures, and CAS is an attractive endovascular approach, less invasive than CEA. Although similar long-term efficacy of CAS and CEA has been demonstrated in asymptomatic patients ^6^ or those younger than 70 years,^7^,^8^ there is concern about the worse result of CAS in elderly patients ^7^ and a greater number of minor strokes in the perioperative period of asymptomatic patients.^6^ Moreover, despite the benefits related to reduced surgical trauma in CAS, this endovascular technique carries a higher risk of hemodynamic depression, when compared to CEA.^9^ The intrinsic risk of Hemodynamic Depression (HD), characterized by postoperative hypotension or bradycardia, is not encompassed by any of the traditional surgical risk indexes.^10^

There is marked heterogeneity in definitions of HD. Criteria might consider absolute values, usually Systolic Blood Pressure (SBP) < 90 mmHg and Heart Rate (HR) < 60 bpm;^11^,^12^ relative values, comparing pre and post-operative vital signs;^13^ and need for treatment.^14^ Independently from the definition adopted, prolonged HD has been associated with a higher risk of perioperative stroke, myocardial infarction, and death after carotid surgery.^15^

The present study aims to add information to this subject by clustering some of the definitions commonly considered, as well as investigating factors associated with HD development and its possible postoperative repercussions.

## Materials and methods

### Objectives

The primary endpoints of the current study are to analyze the prevalence of HD and to identify its predictors. The secondary endpoint is to explore the association between HD and Major Adverse Cardiovascular Event (MACE).

### Study population

The authors performed a retrospective analysis of 254 carotid surgeries performed in a tertiary and a quaternary hospital from January 2014 to December 2018. Missing data determined the exclusion of 17 procedures, effectively resulting in a cohort of 237 procedures. Local ethics committees approved the protocol and written informed consent was waived due to the study's retrospective nature.

### Definitions

Intraoperative hypotension was defined as any of the following, during the surgical procedure: Systolic blood pressure (SBP) < 90 mmHg; Mean arterial pressure (MAP) < 60 mmHg; or need for continuous infusion of vasopressors (norepinephrine). Blood pressure was monitored invasively (arterial line), and data was recorded every 5 or 10 minutes.

Any of the following occurrences in the first 24h characterized postoperative hypotension in an intensive care unit: SBP < 90 mmHg; the need for continuous infusion of vasopressors; or a drop in SBP > 20% compared to preoperative mean ward SBP values on the day before surgery. Blood pressure was monitored continuously and recorded every 1 or 2 hours, and vasopressor infusions were titrated accordingly when needed.

Bradycardia was defined as HR < 50 bpm either intra or post-operatively. Hemodynamic Depression (HD) was the manifestation of either bradycardia or hypotension during the first 24h after surgery and was considered persistent when present in at least two consecutive records.

Symptomatic carotid stenosis was defined as a neurologic deficit directly attributable to the carotid stenosis itself, such as amaurosis fugax, transient ischemic attack, or stroke that occurred up to 6 months before surgical intervention.

MACE was defined as stroke, myocardial infarction, or cardiovascular death, during the hospital stay after surgery. The diagnoses of stroke in the postoperative period were based on the new focal deficits in the patient's clinical evaluation, corroborated by neuroimaging with compatible findings. Myocardial infarction diagnosis was based upon the criteria of a rise and/or fall of troponin, with at least one value above the 99^th^ percentile, and at least one of the following: symptoms of acute myocardial ischemia; new ischemic ECG changes; development of pathological Q waves; imaging evidence of new loss of viable myocardium, or new regional wall motion abnormality in a pattern consistent with an ischemic etiology.^16^ Cardiovascular death was considered when the cause of death was stroke or myocardial infarction.

### Biochemical analysis

All laboratory analyses were ordered at the discretion of the assistant team. Preoperative values correspond to those available most immediately before surgery and no older than 30 days. Postoperative dosages were recorded as the first blood test immediately after surgery in the intensive care unit. Particularly in relation to Troponin T, all samplings up to 120h after surgery were recorded. The Troponin assay used was a high sensitivity Troponin T (Elecsys, Roche Diagnostics, Mannheim, Germany) with a 99^th^ percentile upper reference limit of 0.014 ng/mL.^17^

### Statistical analyses

Variables were tested for normality of distribution using Kolmogorov-Smirnov and Shapiro-Wilk tests. Normally distributed variables are expressed as mean ± Standard Deviation (SD) and were compared using the Student's *t*-test. Variables not fitting normal distribution were expressed as the median and Interquartile Range (IQR) and were compared using the Mann-Whitney *U* test. Chi-Square and Fisher's exact tests were used for categorical data. For the multivariate analysis, different logistic binary regression models comprising variables associated with HD development were compared. The final model was selected based on better Goodness of Fit according to Akaike Information Criterion (AIC), Bayesian Information Criterion (BIC), and Hosmer-Lemeshow. Statistical significance was set at an alpha level of 0.05. Analyses were executed on SPSS for Windows, v26.0.0.0, 64 bits.

## Results

From 254 carotid surgeries performed between January 2014 and December 2018, core data was unavailable for 17 procedures, and 237 procedures were included in the present analysis. Out of the total 237 carotid surgical interventions performed on 220 patients, 178 (75.1%) were Endarterectomies (CEA), and 59 (24.9%) were CAS. Hemodynamic Depression occurred in 54.4% of all procedures. Hypotension took place in 50.2%, was considered persistent in 24.5%, and required a continuous infusion of vasopressors in 8%, bradycardia in 11.0%, persistent in 5.5%, and both hypotension and bradycardia happened in 6.8%.

Overall, 62.0% were male; the mean age was 68.7 years; 31.2% were treated for symptomatic carotid disease and 34.6% had coronary artery disease. In the group that developed HD, the authors found fewer symptomatic patients (24.8% vs. 34.9% in a non-HD group; p = 0.02) and slightly higher mean preoperative SBP (129.37 ± 1.25 vs. 125.52 ± 1.34 in a non-HD group; p = 0.037). There were no other significant preoperative differences between patients with and without HD, regarding clinical, laboratorial, or cardiovascular medications in use (Table 1).

**Table 1:** Preoperative variables vs. hemodynamic depression.

		Hemodynamic Depression			
		HD+	HD-	Global	p
n (%)	Avail.^a^	129 (54.4%)	108 (45.6%)		
**Preoperative**					
Sex, Male	100%	80 (62.0%)	67 (62.0%)	62.0%	**0.997**
Age, years	100%	68.0 (12)	69.0 (13)	68.66	**0.597**
ASA Physical Status	93.24%				**0.122**
II		31 (26.5%)	26 (25%)	24.1%	
III		79 (67.5%)	77 (74.0%)	65.8%	
IV		7 (6.0%)	1 (1.0%)	3.4%	
SBP, mmHg	99.57%	129.37 ± 1.25	125.52 ± 1.34	127.62 ± .92	**0.037**
DBP, mmHg	99.57%	73.7 (13)	71.00 (11)	72.3 (12)	**0.061**
HR, bpm	99.57%	68.96 ± 0.96	69.40 ± .99	68.61 ± .69	**0.301**
Contralateral stenosis	100%	41 (31.8%)	27 (25.0%)	28.7%	**0.250**
Hemoglobin, g/dL	95.78%	13.27 ± 0.17	13.38 ± 0.13	13.34 ± 0.11	**0.626**
Hematocrit, %	95.78%	39.91 ± 0.37	39.62 ± 0.49	39.78 ± 0.30	**0.645**
cTnT, ng/mL	66.66%	0.012 (0.011)	0.011 (0.008)	0.011 (0.008)	**0.108**
Creatinine, mg/dL	93.24%	1.12 (0.42)	1.11 (0.47)	1.12 (0.43)	**0.842**
Hypertension	100%	117 (90.7%)	102 (94.4%)	92.4%	**0.278**
DM	100%	53 (41.1%)	47 (43.5%)	42.2%	**0.706**
Neck radiotherapy	100%	3 (2.3%)	1 (0.9%)	1.7%	**0.628**
COPD	100%	4 (3.1%)	5 (4.6%)	3.8%	**0.735**
CHD	100%	47 (36.4%)	35 (32.4%)	34.6%	**0.516**
Symptomatic Stenosis	100%	32 (24.8%)	42 (38.9%)	31,2%	**0.020**
Stroke	100%	57 (44.2%)	58 (53.7%)	48,5%	**0.144**
Myocardial infarction	100%	26 (20.2%)	24 (22.2%)	21,1%	**0.698**
CHF	100%	11 (8.5%)	9 (8.3%)	8,4%	**0.957**
Aspirin use	99.57%	125 (96.9%)	101 (94.4%)	95.4%	**0.341**
Statin use	99.57%	128 (99.2%)	102 (99.0%)	98.7%	**0.894**
Beta-blocker use	99.57%	62 (48.1%)	40 (37.4%)	43.0%	**0.099**
ACEi/ARB use	99.57%	83 (64.3%)	77 (72.0%)	67.5%	**0.212**

Data expressed as mean ± SD for parametric variables and expressed as median (Interquartile Range) for non-normally distributed variables. ACEi, Angiotensin Converting Enzyme inhibitors; ARB, Angiotensin Receptor Blocker; CHD, Coronary Heart Disease; CHF, Congestive Heart Failure; CKD, Chronic Kidney Disease; COPD, Chronic Pulmonary Obstructive Disease; cTnT, Cardiac Troponin T; DBP, Diastolic Blood Pressure; DM, Diabetes Mellitus; HD+, Occurrence of Hemodynamic Depression; HD-, Absence of Hemodynamic Depression; HR, Heart Rate; SBP, Systolic Blood Pressure.

a Availability of data according to total number of procedures (n = 237).

The incidence of HD was significantly higher after CAS procedures (76.3%) than CEA (47.2%). Patients who had intra-operative bradycardia and/or hypotension were more likely to develop post-operative HD (Table 2).

**Table 2:** Intraoperative variables vs. hemodynamic depression.

		Hemodynamic Depression		
		HD+	HD-	p
n (%)	Avail.^a^	129 (54.4%)	108 (45.6%)	
Intra-operative				
CAS/CEA	100%			≤0.001
CAS		45 (34.9%)	14 (13.0%)	
CEA		84 (65.3%)	94 (87.0%)	
Hypotension	99.15%	29 (22.8%)	12 (11.1%)	0.018
Bradycardia	99.15%	52 (40.9%)]	27 (25.0%)	0.010
Hypotension or Bradycardia	99.15%	62 (48.8%)	32 (29.6%)	0.003
Procedure length, min	97.04%	167.5 (89)	177.5 (60)	0.221
Crystalloid administered, mL	100%	2000 (725)	2000 (1000)	0.400

Data expressed as mean ± SD for parametric variables and expressed as median (Interquartile Range) for non-normally distributed variables.

CAS, Carotid Artery Stenting; CEA, Carotid Endarterectomy; HD+, Occurrence of Hemodynamic Depression; HD-, Absence of Hemodynamic Depression.

a Availability of data according to total number of procedures (n = 237).

Individuals who developed Hemodynamic Depression had a higher post-operative cardiac troponin T peak (0.018 ng/mL, IQR 0.019 vs. 0.014 ng/mL, IQR = 0.018; p = 0.044), higher volume of crystalloids administered in the first 24h after the carotid procedure (2599.89 mL ±76.97 vs. 2223.1 mL ± 79.14; p = 0.001) and a lower postoperative hematocrit (35.43% ± 0.36 vs. 36.68% ± 0.44; p = 0.031). HD did not affect the length of hospital stay after surgery (4, IQR 2 vs. 4, IQR = 2; p = 0.153).

**Fig. 1:**
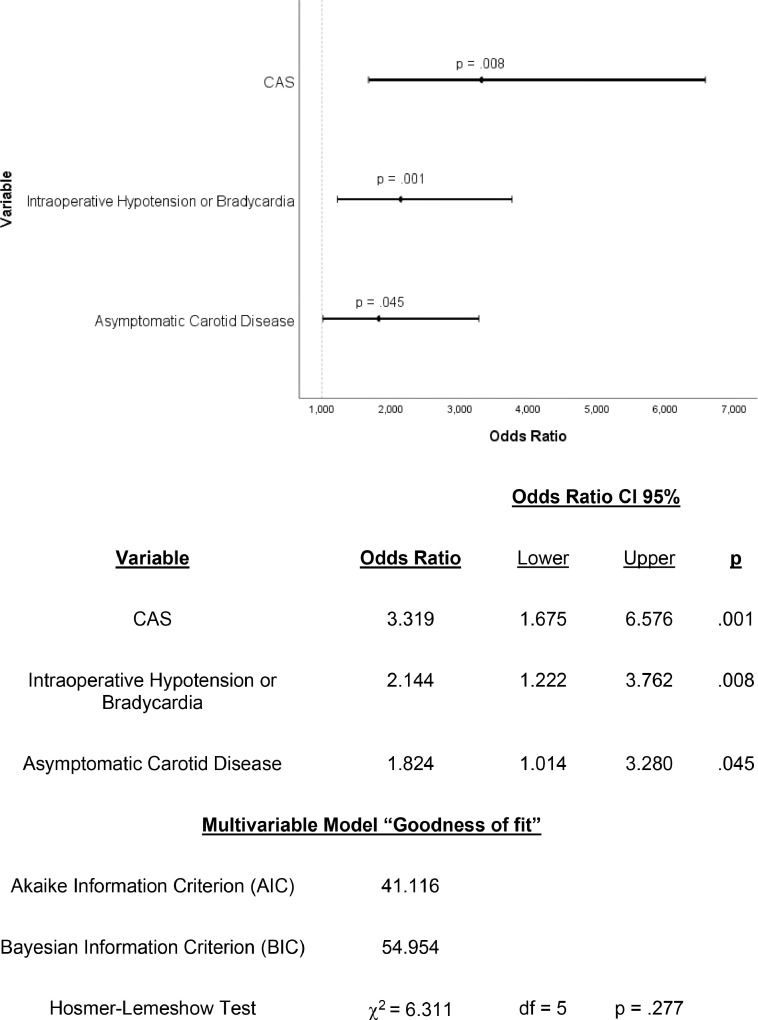
Forest plot – binary logistic regression analysis.

On a binary logistic regression multivariate analysis, asymptomatic carotid stenosis (OR = 1.824; 95% CI 1.014–3.280; p = 0.045), endovascular surgery (OR = 3.319; 95% CI 1.675–6.576; p = 0.001) and intraoperative hypotension or bradycardia (OR = 2.144; 95% CI 1.222–3.762; p = 0.008) were characterized as independent predictors for development of Hemodynamic Depression (Fig. 1).

The authors observed 32 perioperative cardiovascular events in 20 patients (11.0%), distributed as 14 strokes (5.9%), 16 myocardial infarctions (6.8%), and two deaths (0.8%) as a consequence of one stroke and one MI. The incidence of MACE was 13.1% in patients presenting HD and 8.3% in patients without HD (p = 0.298), and in the group of patients with HD and need for continuous vasopressors, there was an independent increase in the risk of MACE, after adjustment for anesthetic technique and procedure laterality (Adjusted Odds Ratio: 5.504; 95% CI 1.729–17.529; p = 0.004).

## Discussion

This study demonstrates that the incidence of HD is high, occurring in more than half of all procedures, and reinforces the potential harm of this condition in its more severe presentation. Endovascular technique, asymptomatic status, and intraoperative hypotension or bradycardia are risk factors for HD.

The occurrence of HD, found to be 54.4% in the whole sample, was higher than previously described, ranging from 7.2%^9^ to 70%^18^ depending on the population, type of procedure performed, and definition adopted for Hemodynamic Depression. In order to capture the full impact of this variable on the studied patients, the authors used broader criteria for HD, encompassing definitions used in other studies, and this might explain the relatively high incidence of HD in the present study.

Following Park et al.,^19^ the authors have identified the surgical technique as an independent predictor of HD, being the endovascular approach associated with a 3.3-fold adjusted incidence of HD in comparison to endarterectomy. A plausible explanation for this fact relies on the baroreflex mechanism: mechanoreceptors distributed along the carotid sinus are overstimulated during angioplasty/stenting, resulting in lower blood pressure and heart rate. The development of bradycardia and hypotension already in the intraoperative setting might work as a marker for this reflex's integrity and sensitivity, potentially indicating patients are at higher risk for HD.

Differently from other studies, diabetes^8^ and prior carotid surgery^20^ were not associated with a lower HD incidence. Moreover, congestive heart failure^9^ and coronary disease were not associated with a higher prevalence of postoperative bradycardia or hypotension in this research.

Perioperative use of Angiotensin-Converting Enzyme (ACE) inhibitors/Angiotensin Receptor Blockers (ARBs) and beta-blockers were not associated with the development of HD in the present study, reinforcing the current recommendation for their perioperative maintenance. Even though beta-blockers can lower heart rate and blood pressure, they should not be discontinued perioperatively,^10^ since withdrawal has been associated with higher mortality after vascular surgery.^21^ Although the use of ACE inhibitors/ARBs has been implicated in the development of hypotension after non-cardiac surgery, there was no difference in the incidence of MACE or death in patients who continued medication perioperatively compared to those who did not use it in a metanalysis,^22^ which is in alignment with the present findings.

A higher incidence of HD was noticed in patients with asymptomatic carotid stenosis. This finding was previously described ^23^ and, therefore, the risk of HD must be thoroughly weighed when considering carotid stenting for asymptomatic individuals. The net clinical benefit of a carotid surgical intervention in asymptomatic patients might not be so evident in comparison to optimized medical therapy alone, and hemodynamic depression can be one of the unexplored reasons for the lack of improvement in event-free survival.^24^

Evidence regarding the association between Hemodynamic Depression and adverse events’ development is conflicting, being supported^9^,^25^ or refuted^15^,^19^ by previous studies. In the present study, hypotension requiring a continuous infusion of vasopressor after surgery was independently associated with the occurrence of MACE. The necessity of vasoactive drugs probably signals a more extreme HD case in which treatment with intravenous crystalloids alone was insufficient to resolve hypotension. Nevertheless, the authors did not detect a greater incidence of MACE in patients who developed HD. Prompt treatment for hemodynamic instability instituted in the intensive care unit, as evidenced by the higher volume of crystalloids administered post-operatively on patients that developed HD, might have attenuated its harmful effect, highlighting the recommendation for perioperative surveillance in the intensive care unit.

There was a positive correlation between HD and elevation of postoperative cTnT. Although previous studies have already demonstrated an association between intraoperative hypotension and a postoperative increase in cTnT,^26^ the present findings must be interpreted with caution, considering that the same association was not observed when the authors considered exclusively acute cTnT elevations, excluding from the analysis patients whose cTnT levels were already elevated before surgery.

This is a retrospective study and, as such, vulnerable to the biases inherent to this type of analysis. Despite being comparable to previous studies,^13^, ^14^, ^15^,^18^,^27^ the sample size might still be considered relatively small to have detected significant differences in the incidence of major complications in the cases of mild HD.

## Conclusions

Incidence of HD after carotid surgery is high and independently associated with the surgical technique, symptomatic repercussion of the carotid stenosis, and intraoperative hypotension or bradycardia. Extreme HD (hypotension requiring the continuous infusion of vasopressors after surgery) was associated with the occurrence of MACE. Therefore, the authors reinforce the need for thorough cardiovascular monitoring in the first 24h after carotid surgery for every patient as a routine, regardless of seemingly at low risk for events because of asymptomatic carotid disease or being treated with a less invasive endovascular technique.

## Authors' contributions

Lauro A. C. Bogniotti: Conceptualization, methodology, validation, formal analysis, investigation, resources, data curation, writing – original draft preparation and creation, project administration. marcelo p. teivelis: validation, investigation, resources, validation, writing - review & editing.

Francisco A. M. Cardozo: Validation.

Bruno Caramelli: Validation, writing ‒ review & editing.

Nelson Wolosker: Validation.

Pedro Puech-Leão: Validation.

Nelson De Luccia: Validation.

Daniela Calderaro: Conceptualization, methodology, formal analysis, investigation, resources, validation, writing ‒ review & editing, supervision, project administration.

## Funding sources

This research did not receive any specific grant from funding agencies in the public, commercial, or not-for-profit sectors.

## Conflicts of interest

The authors declare no conflicts of interest.

## References

[1] Brott TG, Halperin JL, Abbara S (2011). 2011 ASA/ACCF/AHA/AANN/AANS/ACR/ASNR/CNS/SAIP/SCAI/SIR/SNIS/SVM/SVS guideline on the management of patients with extracranial carotid and vertebral artery disease: executive summary: a report of the American College of Cardiology Foundation/American Heart Association Task Force on Practice Guidelines, and the American Stroke Association, American Association of Neuroscience Nurses, American Association of Neurological Surgeons, American College of Radiology, American Society of Neuroradiology, Congress of Neurological Surgeons, Society of Atherosclerosis Imaging and Prevention, Society for Cardiovascular Angiography and Interventions, Society of Interventional Radiology, Society of NeuroInterventional Surgery, Society for Vascular Medicine, and Society for Vascular Surgery. Circulation.

[2] Benjamin EJ, Virani SS, Callaway CW (2018). Heart disease and stroke statistics-2018 update: a report from the American Heart Association. Circulation..

[3] Mantese VA, Timaran CH, Chiu D, Begg RJ, Brott TG, Investigators C. (2010). The Carotid Revascularization Endarterectomy versus Stenting Trial (CREST): stenting versus carotid endarterectomy for carotid disease. Stroke.

[4] Gurm HS, Yadav JS, Fayad P, Katzen BT, Mishkel GJ, Bajwa TK (2008). Long-term results of carotid stenting versus endarterectomy in high-risk patients. N Engl J Med.

[5] Bonati LH, Dobson J, Featherstone RL. (2015). Long-term outcomes after stenting versus endarterectomy for treatment of symptomatic carotid stenosis: the International Carotid Stenting Study (ICSS) randomised trial. Lancet.

[6] Halliday A, Bulbulia R, Bonati LH, Chester J, Cradduck-Bamford A, Peto R (2021). Second asymptomatic carotid surgery trial (ACST-2): a randomised comparison of carotid artery stenting versus carotid endarterectomy. Lancet.

[7] Carotid Stenting Trialists C, Bonati LH, Dobson J (2010). Short-term outcome after stenting versus endarterectomy for symptomatic carotid stenosis: a preplanned meta-analysis of individual patient data. Lancet.

[8] White CJ. (2010). Carotid artery stent placement. JACC Cardiovasc Interv.

[9] Altinbas A, Algra A, Brown MM, Featherstone RL, Kappelle LJ, Jan de Borst G (2014). Effects of carotid endarterectomy or stenting on hemodynamic complications in the International Carotid Stenting Study: a randomized comparison. Int J Stroke.

[10] Kristensen SD, Knuuti J, Saraste A, Anker S, Bøtker HE, De Hert S (2014). 2014 ESC/ESA Guidelines on non-cardiac surgery: cardiovascular assessment and management: the joint task force on non-cardiac surgery: cardiovascular assessment and management of the European Society of Cardiology (ESC) and the European Society of Anaesthesiology (ESA). Eur Heart J.

[11] Gupta R, Abou-Chebl A, Bajzer CT, Schumacher HC, Yadav JS. (2006). Rate, predictors, and consequences of hemodynamic depression after carotid artery stenting. J Am Coll Cardiol.

[12] Bussiere M, Lownie SP, Lee D, Gulka I, Leung A, Pelz DM. (2009). Hemodynamic instability during carotid artery stenting: the relative contribution of stent deployment versus balloon dilation. J Neurosurg.

[13] Cayne NS, Faries PL, Trocciola SM, Saltzberg SS, Dayal RD, Clair D (2005). Carotid angioplasty and stent-induced bradycardia and hypotension: impact of prophylactic atropine administration and prior carotid endarterectomy. J Vasc Surg.

[14] Diehm N, Katzen BT, Dick F, Kovacs M, Zemel G, Powell A (2008). Influence of stent type on hemodynamic depression after carotid artery stent placement. J Vasc Interv Radiol.

[15] Ullery BW, Nathan DP, Shang EK, Wang GJ, Jackson BM, Murphy EH (2013). Incidence, predictors, and outcomes of hemodynamic instability following carotid angioplasty and stenting. J Vasc Surg.

[16] Thygesen K, Alpert JS, Jaffe AS, Chaitman BR, Bax JJ, Morrow DA (2018). Fourth universal definition of myocardial infarction. Circulation.

[17] Puelacher C, Bollen Pinto B, Mills NL, Duceppe E, Popova E, Duma A (2021). Expert consensus on peri-operative myocardial injury screening in noncardiac surgery: a literature review. Eur J Anaesthesiol.

[18] Qazi U, Obeid TE, Enwerem N, Schneider E, White JR, Freischlag JA (2014). The effect of ballooning following carotid stent deployment on hemodynamic stability. J Vasc Surg.

[19] Park BD, Divinagracia T, Madej O, McPhelimy C, Piccirillo B, Dahn MS (2009). Predictors of clinically significant postprocedural hypotension after carotid endarterectomy and carotid angioplasty with stenting. J Vasc Surg.

[20] Lin PH, Zhou W, Kougias P, El Sayed HF, Barshes NR, Huynh TT (2007). Factors associated with hypotension and bradycardia after carotid angioplasty and stenting. J Vasc Surg.

[21] Shammash JB, Trost JC, Gold JM, Berlin JA, Golden MA, Kimmel SE. (2001). Perioperative beta-blocker withdrawal and mortality in vascular surgical patients. Am Heart J.

[22] Hollmann C, Fernandes NL, Biccard BM. (2018). A systematic review of outcomes associated with withholding or continuing angiotensin-converting enzyme inhibitors and angiotensin receptor blockers before noncardiac surgery. Anesth Analg.

[23] Lavoie P, Rutledge J, Dawoud MA, Mazumdar M, Riina H, Gobin YP. (Nov 2008). Predictors and timing of hypotension and bradycardia after carotid artery stenting. AJNR Am J Neuroradiol.

[24] Aboyans V, Ricco JB, Bartelink MEL (2018). 2017 ESC Guidelines on the Diagnosis and Treatment of Peripheral Arterial Diseases, in collaboration with the European Society for Vascular Surgery (ESVS): document covering atherosclerotic disease of extracranial carotid and vertebral, mesenteric, renal, upper and lower extremity arteriesEndorsed by: the European Stroke Organization (ESO)the task force for the diagnosis and treatment of peripheral arterial diseases of the European Society of Cardiology (ESC) and of the European Society for Vascular Surgery (ESVS). Eur Heart J.

[25] Cieri E, De Rango P, Maccaroni MR, Spaccatini A, Caso V, Cao P. (2008). Is haemodynamic depression during carotid stenting a predictor of peri-procedural complications?. Eur J Vasc Endovasc Surg.

[26] van Waes JA, van Klei WA, Wijeysundera DN, van Wolfswinkel L, Lindsay TF, Beattie WS. (Jan 2016). Association between intraoperative hypotension and myocardial injury after vascular surgery. Anesthesiology.

[27] Altinbas A, Algra A, Bonati LH, Brown MM, Kappelle J, Borst GJ (Jan 2014). Periprocedural hemodynamic depression is associated with a higher number of new ischemic brain lesions after stenting in the International Carotid Stenting Study-MRI Substudy. Stroke.

